# Transcriptome and miRNA analyses of the response to *Corynespora cassiicola* in cucumber

**DOI:** 10.1038/s41598-018-26080-6

**Published:** 2018-05-17

**Authors:** Xiangyu Wang, Di Zhang, Na Cui, Yang Yu, Guangchao Yu, Haiyan Fan

**Affiliations:** 1 College of Horticulture, Shenyang Agricultural University, Shenyang, 110866 China; 2 College of Bioscience and Biotechnology, Shenyang Agricultural University, Shenyang, 110866 China; 3 Key Laboratory of Protected Horticulture of Ministry of Education, Shenyang Agricultural University, Shenyang, 110866 China

## Abstract

Cucumber (*Cucumis sativus* L.) target leaf spot (TLS), which is caused by the fungus *Corynespora cassiicola* (*C. cassiicola*), seriously endangers the production of cucumber. In this assay, we performed comprehensive sequencing of the transcriptome and microRNAs (miRNAs) of a resistant cucumber (Jinyou 38) during *C. cassiicola* inoculation using the Illumina NextSeq 500 platform. The possible genes related to the response to *C. cassiicola* were associated with plant hormones, transcription factors, primary metabolism, Ca^2+^ signaling pathways, secondary metabolism and defense genes. In total, 150 target genes of these differentially expressed miRNAs were predicted by the bioinformatic analysis. By analyzing the function of the target genes, several candidate miRNAs that may be related to the response to *C. cassiicola* stress were selected. We also predicted 7 novel miRNAs and predicted their target genes. Moreover, the expression patterns of the candidate genes and miRNAs were tested by quantitative real-time RT-PCR. According to the analysis, genes and miRNAs associated with secondary metabolism, particularly the phenylpropanoid biosynthesis pathway, may play a major role in the resistance to *C. cassiicola* stress in cucumber. These results offer a foundation for future studies exploring the mechanism and key genes of resistance to cucumber TLS.

## Introduction

Cucumber (*Cucumis sativus* L.) is an important greenhouse product. The pathogenic fungus *Corynespora cassiicola* (*C. cassiicola*) can cause cucumber target leaf spot (TLS), which is a fungal disease that occurs worldwide^1^. This fungus is an obligate oomycete pathogen capable of infecting many economically important crops, such as cucumber, tomato, soybean, papaya and cowpea^2^. Currently, studies investigating TLS mainly focus on the identification and control, biological characteristics, genetic laws and molecular markers of resistance genes; however, reports regarding the resistance mechanism in cucumber are scarce.

The process involved in plant responses to stress is extremely complex, involving the coordination of many genes, and because conventional research methods are often confined to the structure and function of genes, exploring the interactions among genes and genetic mechanisms is challenging. Transcriptome sequencing is an important approach for gene expression level analysis, differential expression gene screening, functional gene mining and genetic evolution analysis^3,4^.

Transcriptome sequencing not only assesses changes in the expression level of each transcript under different conditions but also enables the mapping of the defense pathway. Recently, many transcriptome sequencing studies have been performed in plants, including tea, cabbage, banana, tobacco, cucurbits, and cucumber, exploring fungal infections^5–10^. Plant hormone signaling pathways, transcription factors, protein kinases and pathogenesis-related (PR) genes play important roles in plant disease resistance.

MiRNAs are a class of non-coding single stranded RNA molecules encoded by endogenous genes, and their main function is to participate in gene expression and regulation at the post-transcriptional level^11^. Plant miRNAs were first obtained from *Arabidopsis thaliana* small RNA libraries, and thousands of plant miRNAs are currently registered in the miRBase sequence database (http://www.mirbase.org/). Plant miRNAs have a variety of biological functions that may be involved in the regulation of plant growth and development, hormone signal transduction and environmental stress responses^12^. MiRNAs in plants have two mechanisms of action. First, plant miRNAs are similar to small interfering RNAs (siRNAs); plant miRNAs are perfectly matched to target mRNAs, miRNAs have 5’ terminal residues that can be identified with the open reading frame (ORF) of the target mRNA, and miRNAs are completely complementary for cleavage^13^. Second, the combination of plant miRNAs and target mRNA is not fully matched; thus, mRNA translation is inhibited but does not affect transcription^14^. The target genes of miRNAs in plants are mostly transcription factors involved in plant growth and the response to environmental stresses. Therefore, miRNAs play a vital role in plant development and the adaptation to adverse situations^15,16^. Certain plant miRNAs can down-regulate the expression of target genes to cope with the demands of growth and environmental stress”. Down-regulation is a very complex process, and a feedback inhibition regulation pathway has been proposed to exist among the miRNAs and target genes^17,18^.

Many miRNAs in plants can be induced following pathogen infections; these miRNAs can interfere with the expression of genes by interacting with target genes^19^. In *Arabidopsis thaliana*, a significant difference was observed in the expression levels of miR156, miR159, miR166, miR825, miR852 and miR843 after *Pseudomonas syringae* infection^20^. In *Verticillium dahliae*-inoculated cotton roots, 65 miRNA families exhibited differences in expression^21^. In Chinese wild grape, researchers have identified miRNAs that may be involved in powdery mildew resistance^22^. Similarly, under powdery mildew stress, 79 miRNAs were found in wheat leaves^23^. In cucumbers, some researchers have identified a few miRNAs associated with the development and response to stress^24–28^. However, there are no reports of miRNAs associated with the response to *C. cassiicola*.

In this study, high-throughput sequencing was performed to investigate the changes in the transcriptome and miRNAs in cucumber infected with *C. cassiicola*. This assay screened for differentially expressed genes (DEGs) and found miRNAs involved in the response to disease stress in cucumber, providing a new theoretical basis for studies investigating disease resistance mechanisms and key resistance genes in TLS.

## Results

### Pathogen invasion and plant response

**Figure 1:**
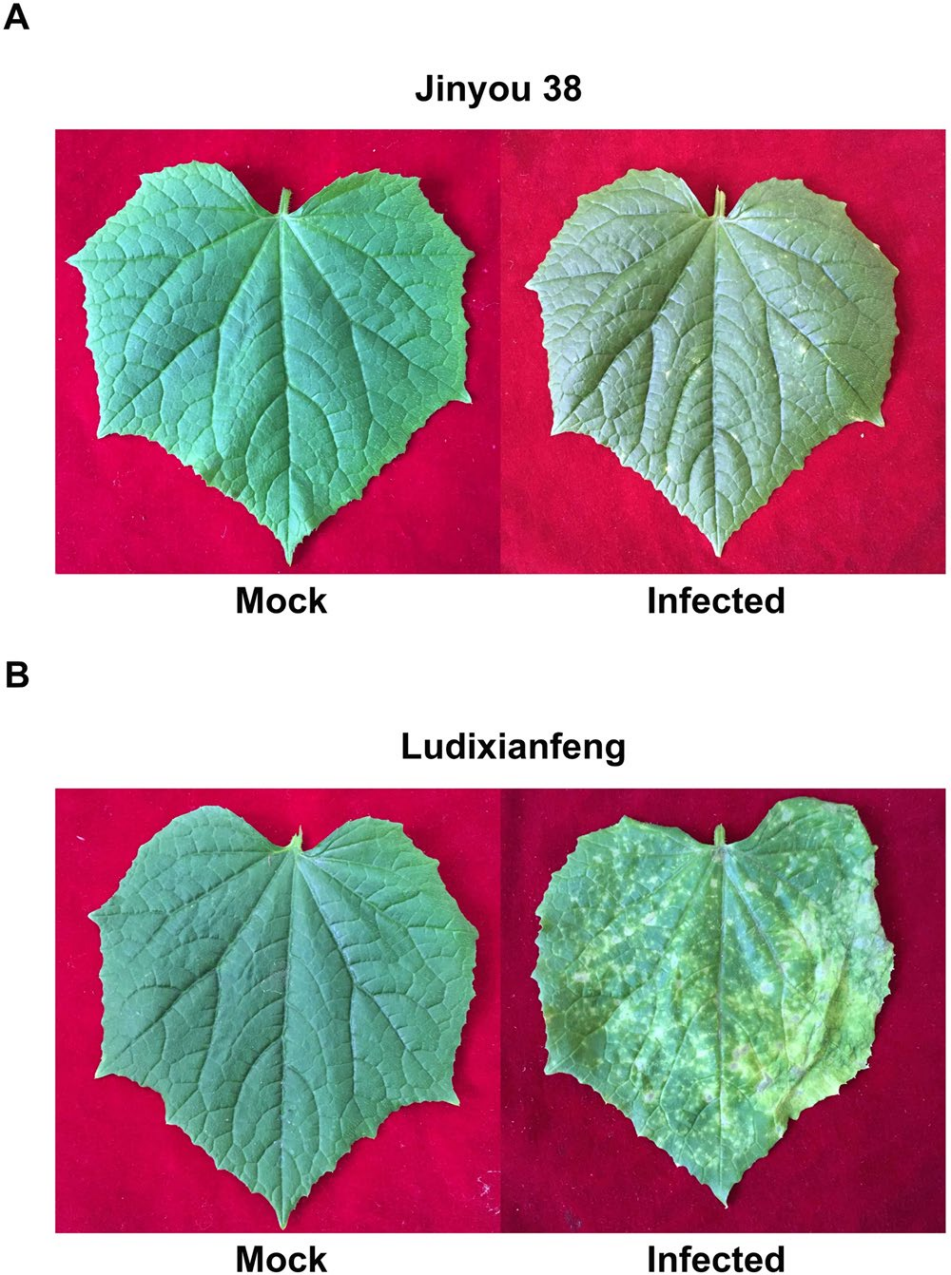
Symptoms of *C. cassiicola* infection of Jinyou 38 (resistant) and Ludixianfeng (susceptible) cucumber. (**A**) Jinyou 38 infected with *C. cassiicola* for 0 days and 5 days, respectively. (**B**) Ludixianfeng infected with *C. cassiicola* for 0 days and 5 days, respectively.

**Figure 2:**
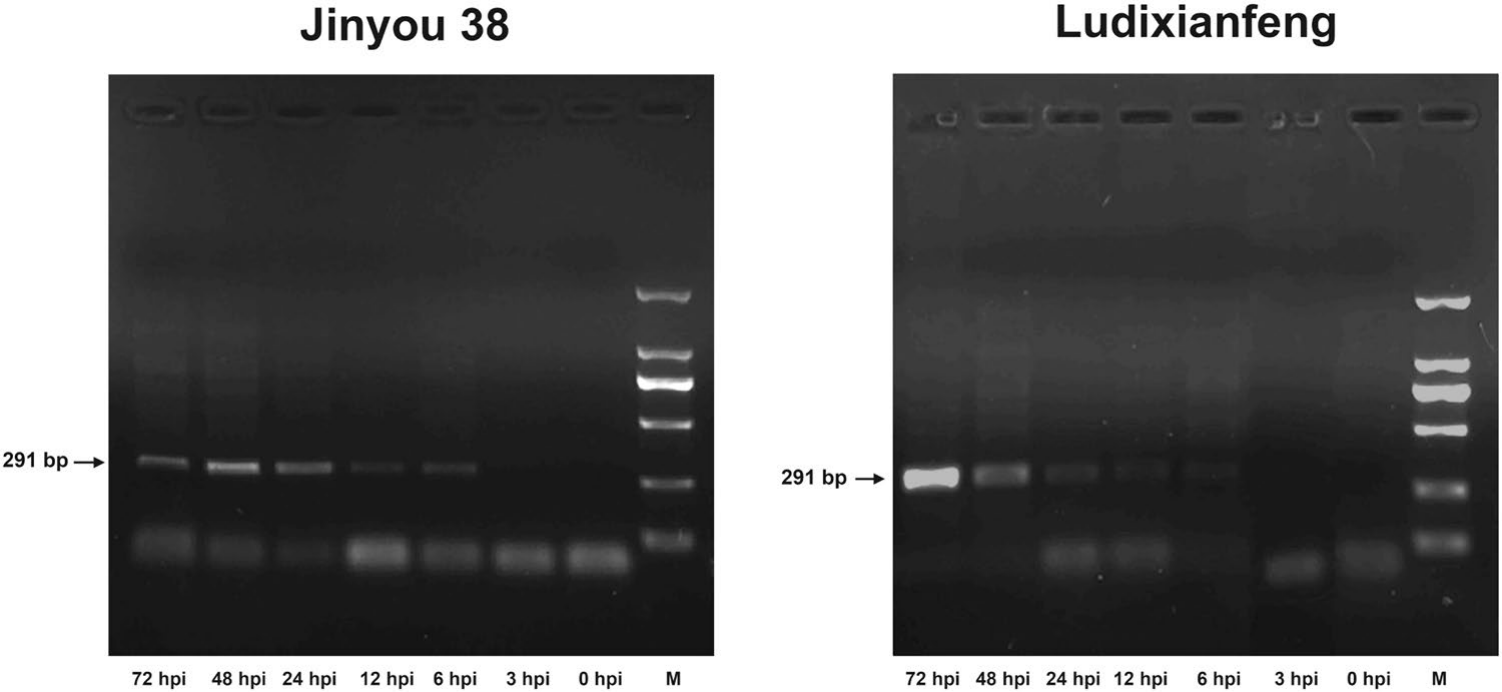
PCR detection of pathogen invasion using DNA primers specific for the *C. cassiicola* internal transcribed space. The samples were derived from the same experiment and that the gels were processed in parallel. The results for the Jinyou 38 resistant variety are shown in the gel on the left, and the results for the Ludixianfeng susceptible variety are shown in the gel on the right. M = DL 2000 DNA marker.

**Figure 3:**
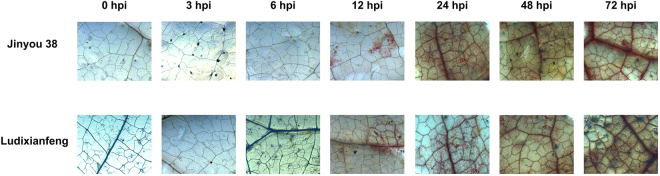
Phenotype changes of lignin in leaves of cucumber after *C. cassiicola* treatment. Magnification 40X.

**Figure 4:**
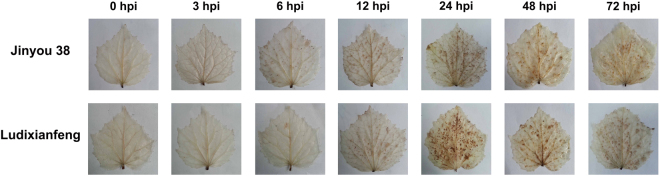
Phenotype changes of H_2_O_2_ in leaves of cucumber after *C. cassiicola* treatment.

**Figure 5:**
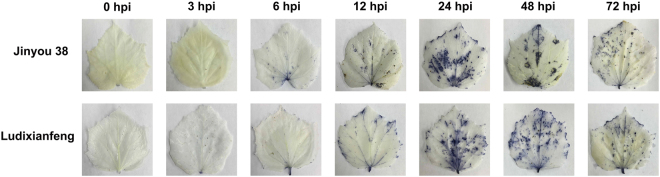
Phenotype changes of O_2˙_^−^ in leaves of cucumber after *C. cassiicola* treatment.

After inoculation with *C. cassiicola*, resistant (Jinyou 38) and susceptible (Ludixianfeng) cucumber varieties were investigated to detect changes in phenotypes and lesions (Fig. 1). The symptoms of the resistant variety were mild (Fig. 1A). Compared with the resistant variety, the susceptible variety showed more severe symptoms (Fig. 1B). Because the ITS fungal sequence is specific, we used PCR to quickly detect pathogen invasion; the amplified fragment was 291 bp. Approximately 6 h post-inoculation (hpi), the ITS sequence of the pathogen was amplified in the both varieties by PCR (Fig. 2). As shown in Fig. 3, after inoculation with *C. cassiicola*, the red complex began to appear in both varieties of cucumber leaves at 12 hpi, which indicated that lignin was deposited, and the lignin accumulation increased over time. At 12–24 hpi, staining of the resistant variety was stronger than that of the susceptible variety. This finding indicated that the rate of lignin production in the resistant variety was faster under *C. cassiicola* stress than that of the susceptible variety. As shown in Fig. 4, brown spots began to appear in the resistant and susceptible cucumber varieties at 6 hpi, which indicated that H_2_O_2_ began to accumulate and gradually increase in the leaves. At 24 hpi, a large area in the leaves was stained, showing that the leaves accumulated a higher level of H_2_O_2_. However, at 48 hpi, the accumulation of H_2_O_2_ showed a decreasing trend. During the early stage of the pathogen treatment (6–12 hpi), the staining in the leaves of the resistant variety was deeper, indicating that the outbreak of H_2_O_2_ in the resistant variety was stronger than that in the susceptible variety. However, at 24 hpi, compared with the resistant cultivar, the susceptible cultivar showed leaves of a deeper color and greater H_2_O_2_ accumulation. In the NBT staining experiment, blue spots began to appear in both cucumber varieties at 6 hpi, and large areas of blue began to appear in both varieties at 24 hpi; however, at 48 hpi, the O_2_^.−^ accumulation began to gradually decrease. Similarly, during the early stages of infection, the accumulation rate of O_2_^.−^ was faster and the accumulation was larger in the resistant variety (Fig. 5). The results of PCR detection showed that *C. cassiicola* could be detected in both varieties at 6 hpi. Meanwhile, H_2_O_2_ and O_2_^.−^ began to accumulate at 6 hpi, but the accumulation in the resistant variety was stronger. At 24 hpi, accumulation of lignin, H_2_O_2_ and O_2_^.−^ in both varieties began to increase significantly. In summary, the resistant variety can respond to stress faster after *C. cassiicola* infections. Thus, we selected samples of the resistant variety from 0, 6 and 24 hpi for high-throughput sequencing.

### Gene mapping and functional annotation

According to the transcriptional sequencing, 30 764 714, 33 175 550 and 27 825 692 clean reads were obtained from the leaves at time point 1 (0 hpi), time point 2 (6 hpi), and time point 3 (24 hpi), respectively. The reads significantly concentrated in scaffold00542, scaffold02229 and scaffold03356 (Supplementary Table S1). All quality reads were assembled and annotated using BLAST against the cucumber genome database.

### eggNOG analysis of functional categories

**Figure 6:**
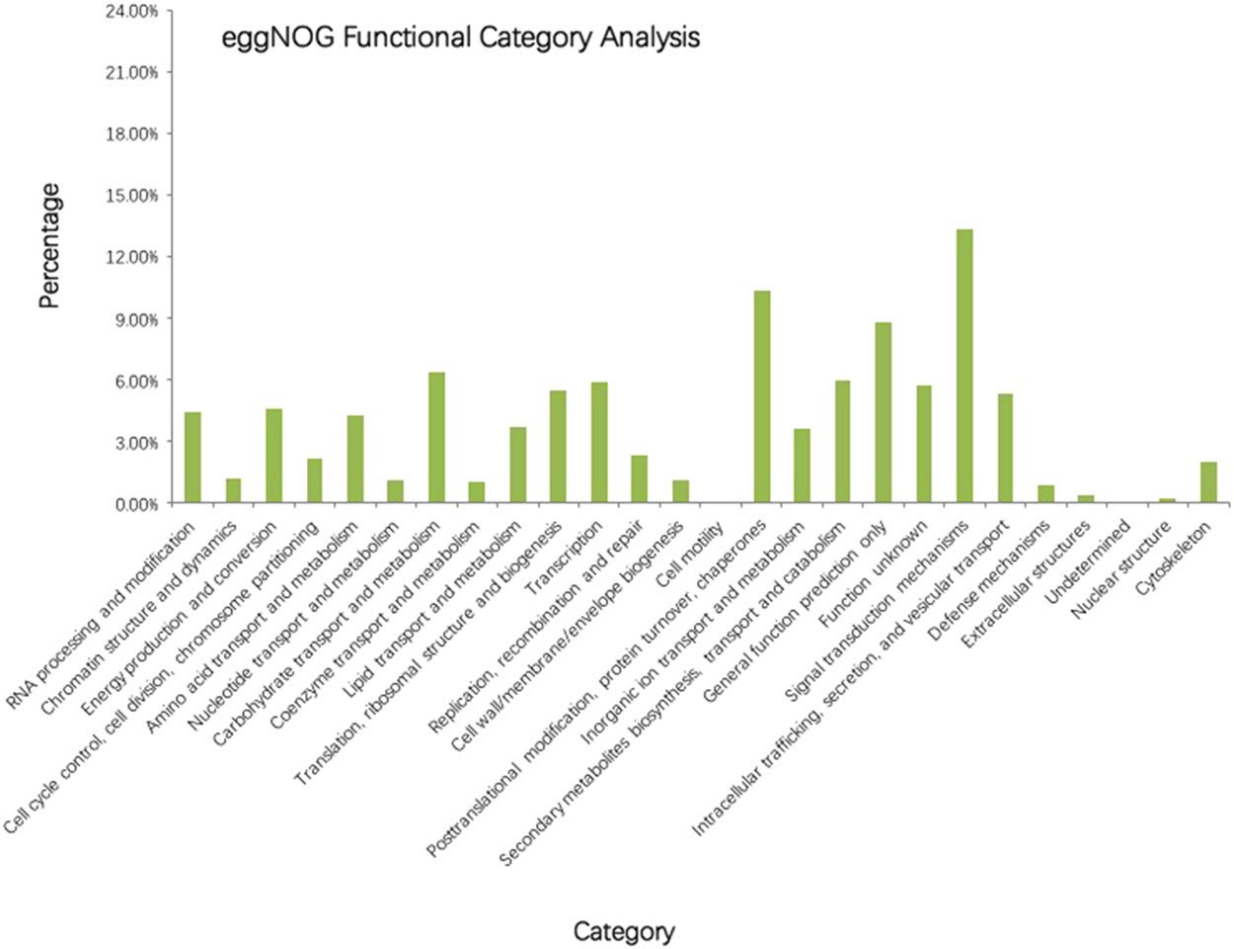
eggNOG analysis of functional category.

The eggNOG database can cluster eukaryotic orthologous proteins. The annotated genes were searched in the eggNOG database to determine their functional classification. In total, 9527 genes were classified into 26 categories according to eggNOG. The largest group was “signal transduction mechanisms” (1269, 13.32%), followed by “posttranslational modification, protein turnover, chaperones” (988, 10.37%), “general function prediction only” (839, 8.81%), “carbohydrate transport and metabolism” (605, 6.35%) and “secondary metabolite biosynthesis, transport, and catabolism (569, 5.97%)” (Fig. 6).

### Analysis of DEGs

In total, 21 503 genes were reviewed, and pairwise comparisons were performed between each time point (0 hpi vs 6 hpi and 6 hpi vs 24 hpi). Genes with at least a twofold change were considered DEGs (*P*-value < 0.05).

**Figure 7:**
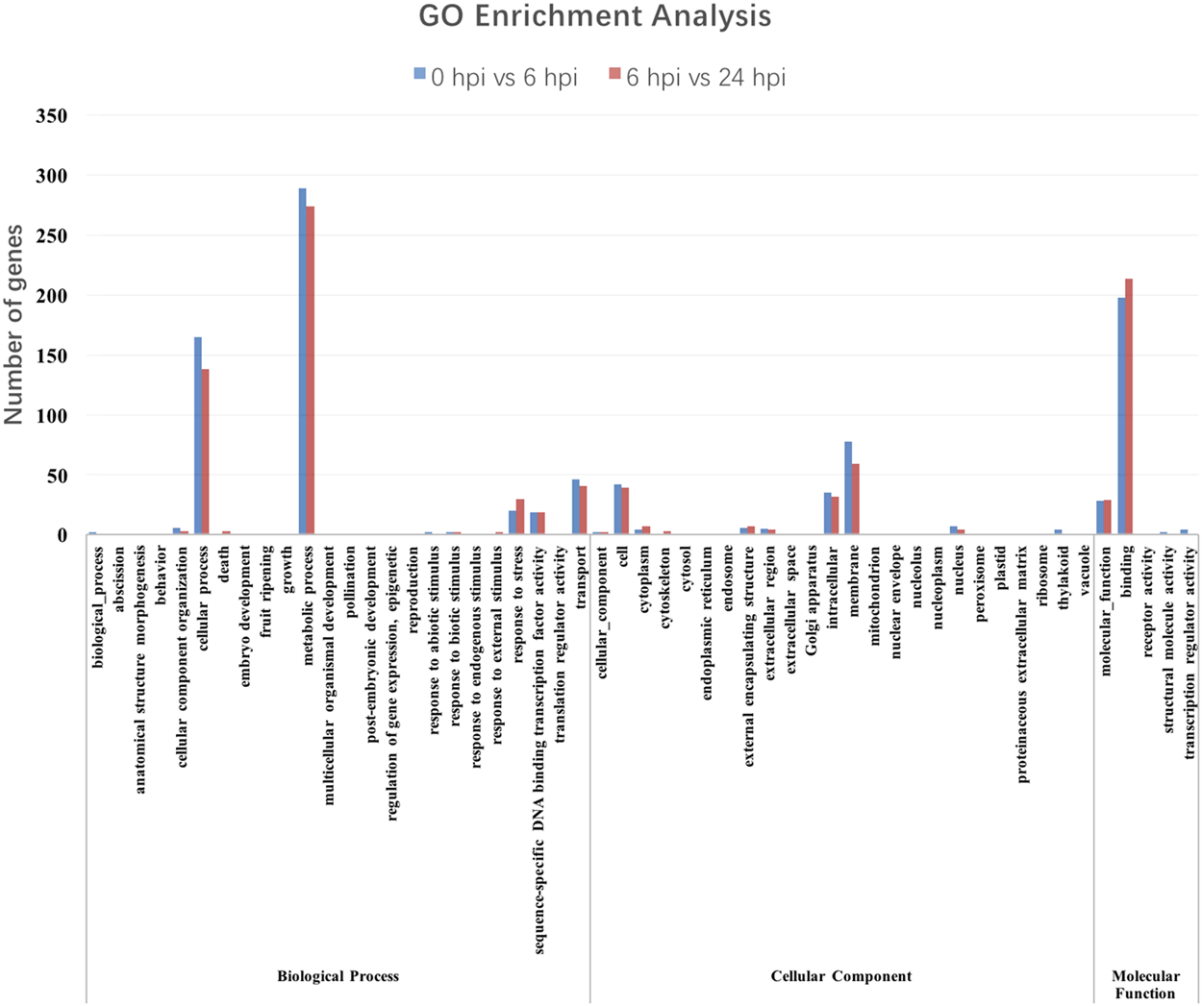
Gene ontology enrichment analysis of differentially expressed genes.

To further explore the functions of the DEGs, we performed a Gene Ontology (GO) enrichment analysis (Fig. 7). The GO terms of the DEGs were significantly enriched in “metabolic process”, “response to stress”, “transport”, “extracellular region” and “molecular function” (Table 1).

**Table 1:** Significantly enriched GO terms (P < 0.05).

Gene Ontology analysis	P value 6 hpi/0 hpi	P value 24 hpi/6 hpi
metabolic process	1.62E-05	5.59E-06
response to stress	0.003606627	3.63E-08
transport	0.003696542	0.01210922
extracellular region	0.009152109	0.03206361
membrane	1.80E-05	0.0200275
molecular function	0.000360898	4.67E-05

**Figure 8:**
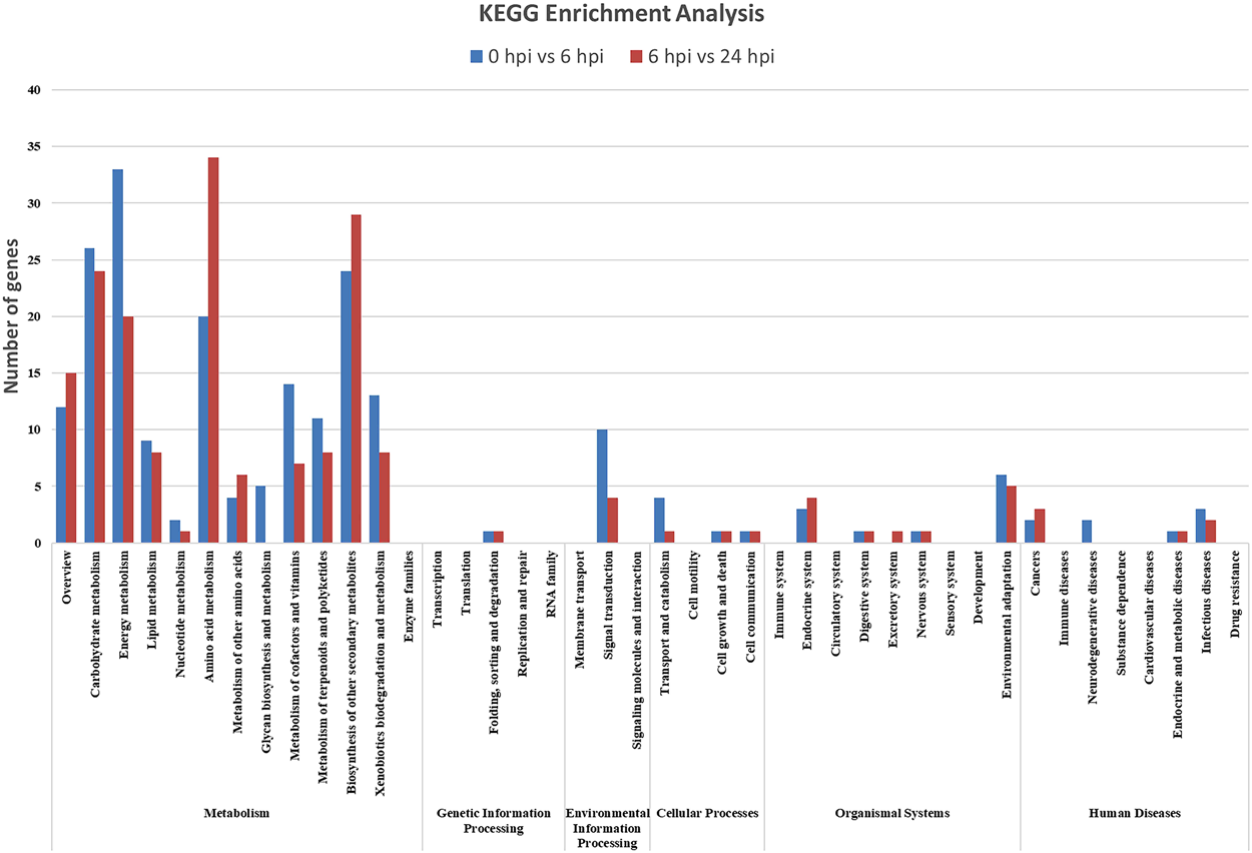
KEGG enrichment analysis of differentially expressed genes.

We also performed a Kyoto Encyclopedia of Genes and Genomes (KEGG) pathway enrichment analysis for the DEGs (Fig. 8). “Energy metabolism”, “amino acid metabolism”, “metabolism of terpenoids and polyketides” and “biosynthesis of other secondary metabolites” were the enriched categories for the DEGs (Table 2).

**Table 2:** Significantly enriched KEGG pathways (P < 0.05).

KEGG pathway	P value 6 hpi/0 hpi	P value 24 hpi/6 hpi
Energy metabolism	1.07E-09	8.72E-04
Amino acid metabolism	2.86E-02	3.18E-08
Metabolism of terpenoids and polyketides	0.001831356	0.02930754
Biosynthesis of other secondary metabolites	2.94E-10	4.76E-15

**Figure 9:**
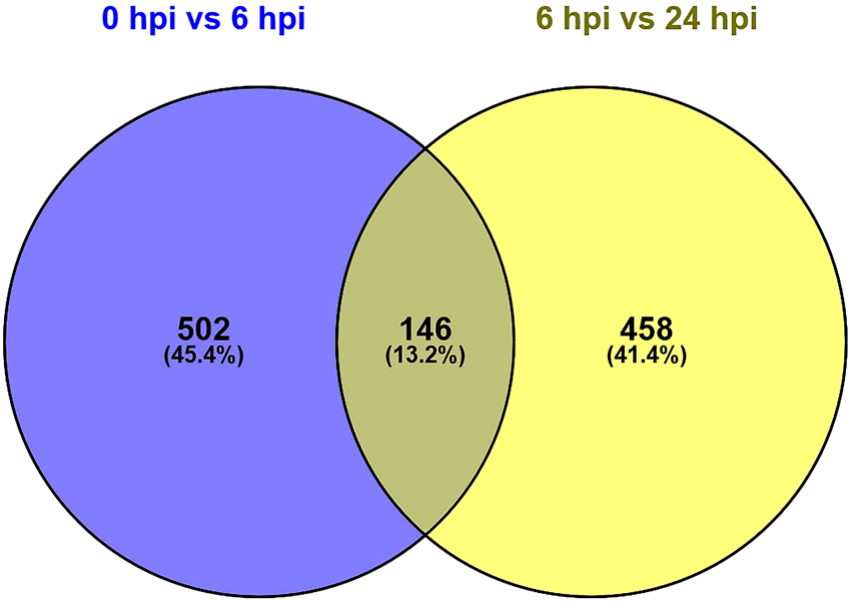
Venn diagram analysis between two differentially expressed gene sets (0 hpi vs 6 hpi; 6 hpi vs 24 hpi).

A Venn diagram analysis was performed on the DEG sets (0 hpi vs 6 hpi and 6 hpi vs 24 hpi) (Fig. 9). We analyzed the intersection of the DEGs between different time points to investigate the DEGs in the resistant variety over time; this analysis further helped our understanding of the response to *C. cassiicola* infection in cucumber. In total, 146 DEGs were included in the intersection, and these genes are currently the focus of our future studies (Supplementary Table S2). According to these experiments, we selected and classified certain candidate genes that may be associated with the response to *C. cassiicola* in cucumber (Table 3).

**Table 3:** Candidate genes may be associated with response to *C. cassiicola* in cucumber.

Category	Gene ID	Gene description
Plant hormone	Cucsa.004570	Gibberellin 3-oxidase
	Cucsa.089960	S-adenosylmethionine decarboxylase
	Cucsa.343030	Gibberellin regulated protein
Transcription factor	Cucsa.121500	MYB transcription factor
Metabolic correlation	Cucsa.147540	Phosphoenolpyruvate carboxylase
	Cucsa.339710	Carbonic anhydrase
Ca^2+^ signaling pathways	Cucsa.254730	Calmodulin-like
Secondary metabolism	Cucsa.017550	Pleiotropic drug resistance protein
	Cucsa.342000	Cytochrome P450 CYP2
	Cucsa.124480	Phenylalanine ammonia-lyase
	Cucsa.261210	4-coumarate: CoA ligase
Defense genes	Cucsa.092350	Xyloglucan endotransglucosylase/hydrolase family protein
	Cucsa.152090	Chitinase
	Cucsa.133220	Pathogenesis-related protein
	Cucsa.302870	Thaumatin
	Cucsa.043900	Aspartyl protease family protein
	Cucsa.153390	Peroxidase

### Verification of candidate genes by qRT-PCR

**Figure 10:**
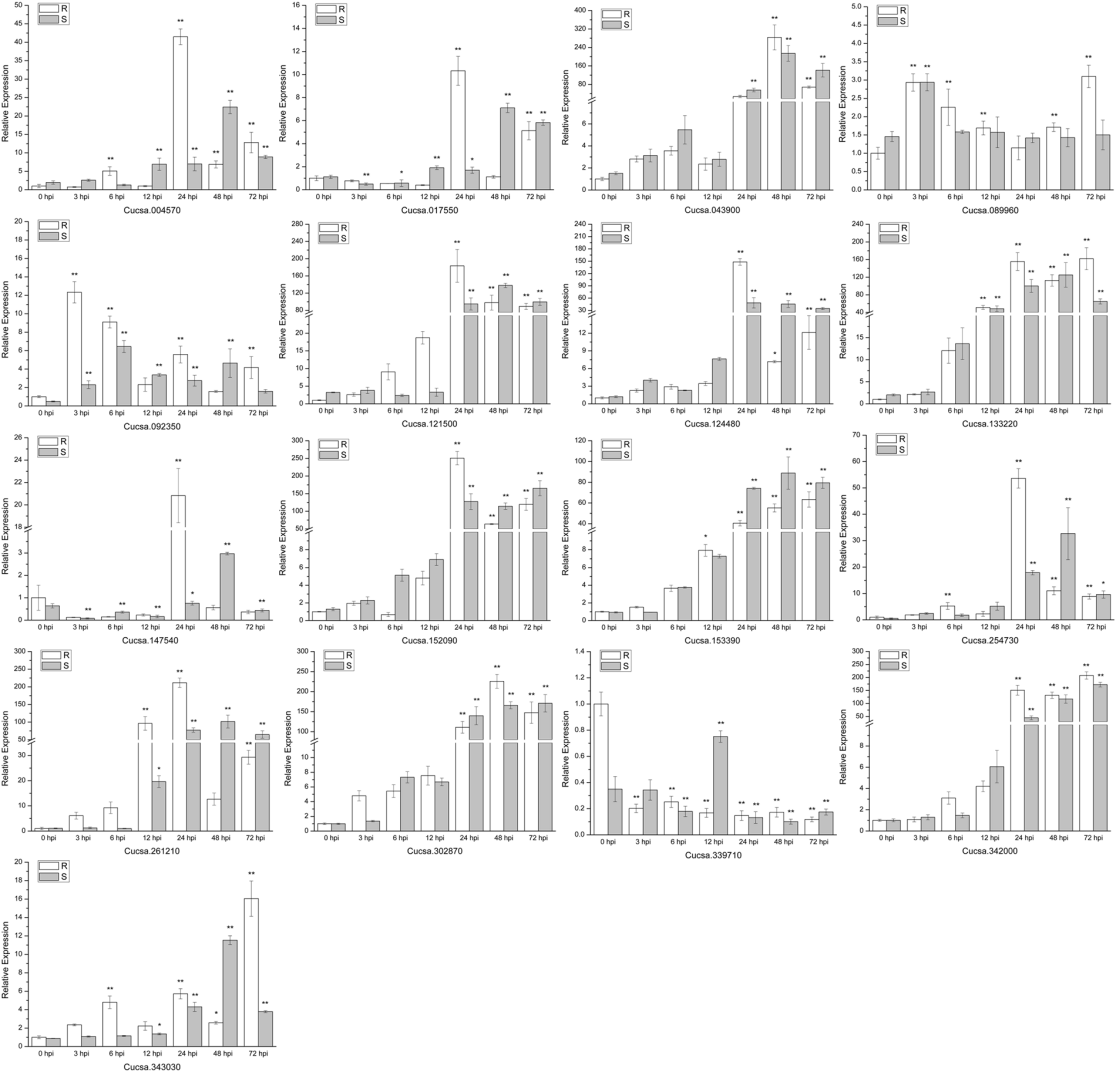
Expression analysis of 17 candidate genes at 0, 3, 6, 12, 24, 48 and 72 hpi (hours post-inoculation) using the 2^−ΔΔCt^ method. Data are means (±SD) of three biological replicates per variety. Significance was determined by Duncan’s multiple range test, and is represented by *(P ≤ 0.05) and **(P ≤ 0.01). R = resistant variety. S = susceptible variety.

To validate the reliability of the transcriptome data and analyze the expression of stress-responsive genes, 17 candidate genes were selected for qRT-PCR assays. We conducted experiments at different time points (i.e., 0, 3, 6, 12, 24, 48 and 72 hpi) using the resistant and susceptible varieties (Fig. 10). The results of the qRT-PCR were consistent with the transcriptome data. Thus, the expression levels of all genes were consistent with the RNA-seq data, indicating that our transcriptome results were reliable for further studies. The expression of *gibberellin 3-oxidase* (Cucsa.004570) in the resistant variety increased at 6 hpi and reached a peak at 24 hpi, but in the susceptible variety, a significant increase was observed at 48 hpi. The expression of *gibberellin regulated protein* (Cucsa.343030) in the resistant variety significantly increased at 6 hpi and remained significantly up-regulated at 72 hpi. In the susceptible variety, the expression of *gibberellin regulated protein* reached a peak only at 48 hpi. The expression of *S-adenosylmethionine decarboxylase* (Cucsa.089960) in both varieties reached a peak at 3 hpi but displayed a rebound in Jinyou 38 at 72 hpi. The expression of *MYB transcription factor* (Cucsa.121500) in the resistant variety was slightly higher than that in the susceptible variety at 6 hpi and 12 hpi; however, the expression of *MYB transcription factor* was significantly up-regulated in both varieties after 24 hpi. The expression of *phosphoenolpyruvate carboxylase* (Cucsa.147540) was slightly down-regulated in both varieties but significantly up-regulated in the resistant variety at 24 hpi and up-regulated in the susceptible variety at 48 hpi. After the infection, the expression of *carbonic anhydrase* (Cucsa.339710) decreased in both varieties. *Calmodulin-like* (Cucsa.254730) showed a significant increase after 6 hpi in Jinyou 38 and obviously increased after 24 hpi in Ludixianfeng. The expression of *pleiotropic drug resistance protein* (Cucsa.017550) in the resistant variety was significantly up-regulated at 24 hpi, but this up-regulation occurred in the susceptible variety at 48 hpi. After 24 hpi, *cytochrome P450 CYP2* (Cucsa.342000) had a high abundance in both varieties. However, in the resistant variety, its accumulation was higher, particularly at 24 hpi. The expression of *phenylalanine ammonia-lyase* (Cucsa.124480) and *4-coumarate: CoA ligase* (Cucsa.261210) in the resistant variety reached a peak at 24 hpi but reached its peak in the susceptible variety at 48 hpi. The expression of *xyloglucan endotransglucosylase/hydrolase family protein* (Cucsa.092350) in Jinyou 38 reached a peak earlier at 3 hpi. After 24 hpi, *chitinase* (Cucsa.152090) maintained a high expression level in both varieties. Similarly, the expression patterns of *PR protein* (Cucsa.133220), *thaumatin* (Cucsa.302870), *aspartyl protease family protein* (Cucsa.043900) and *peroxidase* (Cucsa.153390) in the resistant and susceptible varieties were nearly identical, and after 12–24 hpi, their abundance was at a very high level. The qRT-PCR results showed that secondary metabolism-related genes were strongly induced after the *C. cassiicola* infection. There was no significant difference in the expression of these candidate genes in both varieties at 0 hpi. However, the expression of most of the candidate genes changed significantly at 6–24 hpi. In addition, these genes showed stronger changes in the resistant variety than in the susceptible variety during the early stage of infection. In short, the resistant variety could respond more rapidly to *C. cassiicola* stress and induce more effective defenses.

### Annotations of known miRNAs

To identify the known miRNAs, the clean reads were used in the BLAST search against known mature miRNAs and pre-miRNAs of miRBase (Version 21.0). In total, 64, 61 and 59 miRNAs with a high sequence similarity to known miRNAs were identified at 0 hpi, 6 hpi and 24 hpi, respectively (Table 4). Analyses of the known conserved miRNAs were performed by comparisons to major dicotyledonous species in miRbase (Version 21.0). The results showed that the known miRNAs were relatively conserved in cucumber and *Arabidopsis thaliana* (Supplementary Table S3).

**Table 4:** MiRNAs annotation results statistics.

Sample	miRNAs	Unique reads	Total reads
0 hpi	64	4618	637716
6 hpi	61	5128	917899
24 hpi	59	3535	420995

### Identification of DEMs and their targets

**Figure 11:**
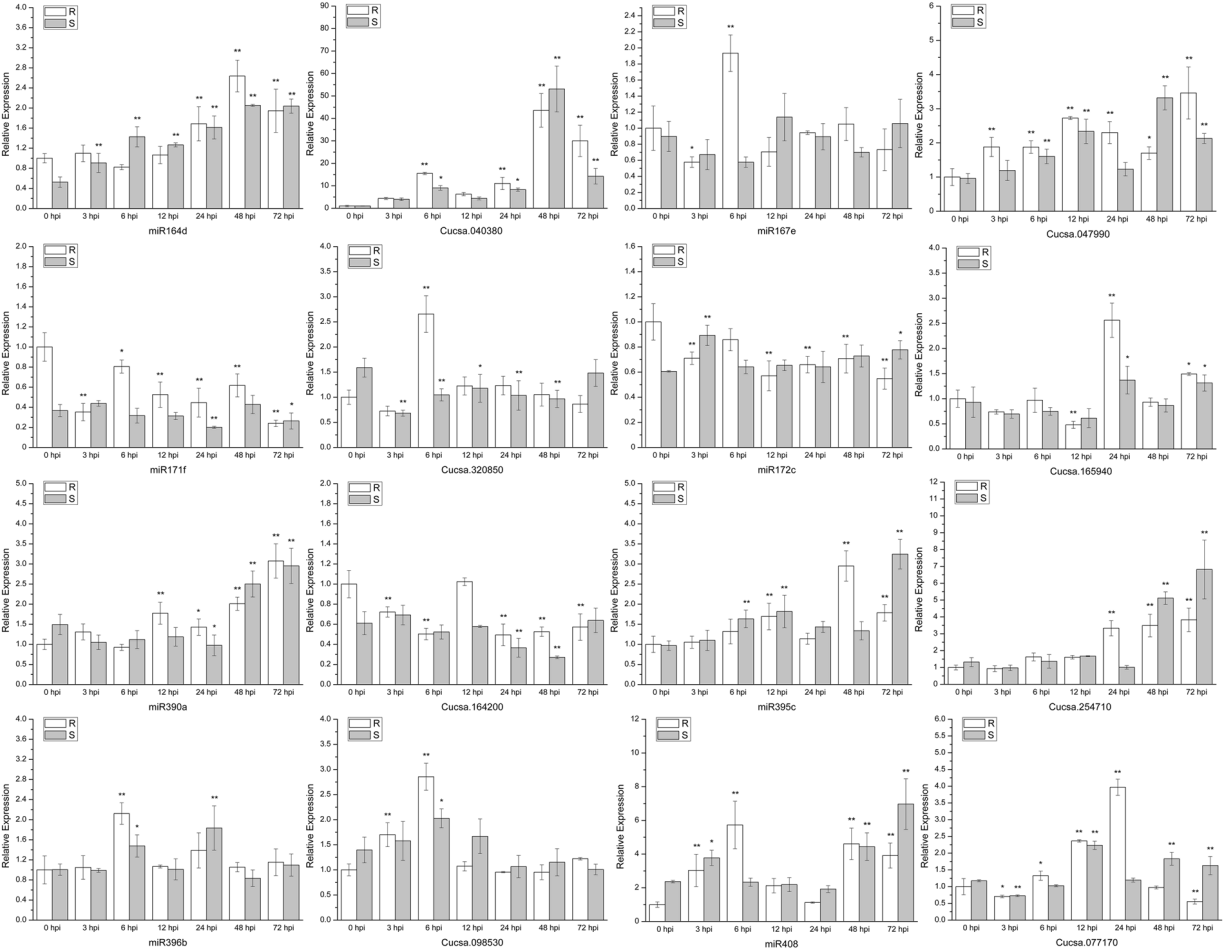
Expression analysis of candidate miRNAs and targets at 0, 3, 6, 12, 24, 48 and 72 hpi (hours post-inoculation) using the 2^−ΔΔCt^ method. Data are means (±SD) of three biological replicates per variety. Significance was determined by Duncan’s multiple range test, and is represented by *(P ≤ 0.05) and **(P ≤ 0.01). R = resistant variety. S = susceptible variety.

According to the difference in expression (|fold change| > 2) in the multi-sample group comparison, we screened the differentially expressed mature miRNAs (DEMs). The miRNAs are primarily bound to target sites by complementary pairing, and we used genomic information and transcriptome data to predict the target genes in cucumber. In total, 150 target genes in 34 DEMs were predicted (Supplementary Table S4). Several miRNAs and their target genes that may be involved in the response to *C. cassiicola* were tested by qRT-PCR at different stages of pathogen invasion in the resistant and susceptible cucumber lines (Table 5). The qRT-PCR results validated that the expression patterns of the miRNAs and target genes were consistent with the RNA-seq data (Fig. 11).

**Table 5:** Candidate miRNAs and their targets.

MicroRNA	Mature sequence	Targets ID	Targets description
miR164d	UGGAGAAGCAGGGCACGUGCA	Cucsa.040380	NAC domain containing protein
miR167e	UCAAGCUGCCAGCAUGAUCUA	Cucsa.047990	Auxin response factor (ARF)
miR171f	UGAUUGAGCCGUGCCAAUAUC	Cucsa.320850	GRAS family transcription factor
miR172c	AGAAUCUUGAUGAUGCUGCAU	Cucsa.165940	Ethylene-responsive transcription factor (APETALA)
miR390a	AAGCUCAGGAGGGAUAGCGCC	Cucsa.164200	Leucine-rich repeat receptor-like protein kinase family protein
miR395c	UUGAAGUGUUUGGGGGAACUC	Cucsa.254710	ATP sulfurylase
miR396b	UUCCACAGCUUUCUUGAACUG	Cucsa.098530	Anthranilate phosphoribosyltransferase
miR408	AUGCACUGCCUCUUCCCUGGC	Cucsa.077170	Plantacyanin

The expression of miR164d and miR395c in both varieties increased noticeably after the pathogen infection. The expression of miR167e was first decreased at 3 hpi but significantly increased at 6 hpi in Jinyou 38. The accumulation of miR171f and miR172c was induced at 3 hpi in Jinyou 38, however, the expression of miR171f and miR172c increased in Ludixianfeng at 3 hpi. The expression of miR390a was significantly increased at 48–72 hpi in both varieties. In the resistant variety, miR396b was markedly up-regulated at 6 hpi, while in the susceptible variety, miR396b obviously increased at 24 hpi. The expression of miR408 obviously increased at 3–6 hpi in Jinyou 38 and was significantly up-regulated at 48–72 hpi in both varieties. Compared with the positive control (0 hpi), the expression of *NAC domain containing protein* (Cucsa.040380), which is cleaved by miR164d, was significantly up-regulated at 6, 24, 48 and 72 hpi in Jinyou 38 and reached a peak at 48 hpi in both varieties. After the pathogen infection, *auxin response factor* (Cucsa.047990), which is cleaved by miR167e, was always increased in both varieties, except for the susceptible variety at 24 hpi. The expression of *GRAS family transcription factor* (Cucsa.320850), which is cleaved by miR171f, was noticeably reduced after the pathogen inoculation in both varieties, while it was markedly up-regulated at 6 hpi in the resistant variety. *Ethylene-responsive transcription factor* (Cucsa.165940), which is targeted by miR172c, significantly decreased at 12 hpi and noticeably increased at 24 hpi in the resistant variety. The expression of *leucine-rich repeat receptor-like protein kinase family protein* (*LRR-RLK*) (Cucsa.164200), which is cleaved by miR390a, was significantly down-regulated after the pathogen infection but recovered to the original level at 12 hpi in the resistant variety; however, *LRR-RLK* was significantly decreased at 24 and 48 hpi in the susceptible variety. *ATP sulfurylase* (Cucsa.254710), which is targeted by miR395c, was significantly increased after 24 hpi in Jinyou 38, while in Ludixianfeng, its expression obviously increased at 48 and 72 hpi. The expression of *anthranilate phosphoribosyltransferase* (Cucsa.098530), which is cleaved by miR396b, noticeably increased at 3 and 6 hpi and then recovered to the normal level in Jinyou 38 but fluctuated slightly after the *C. cassiicola* infection in Ludixianfeng. *Plantacyanin* (Cucsa.077170), which is targeted by miR408, was markedly up-regulated after 6 hpi and gradually decreased in the resistant variety, while in the susceptible variety, its expression fluctuated, and the main increments occurred at 12, 48 and 72 hpi. Similarly, most of the miRNAs and targets changed more significantly in the resistant variety than in the susceptible variety at 6–24 hpi. This finding was also consistent with the previous finding that the resistant variety could respond more rapidly to *C. cassiicola* stress.

### Identification of novel miRNAs and their targets

**Figure 12:**
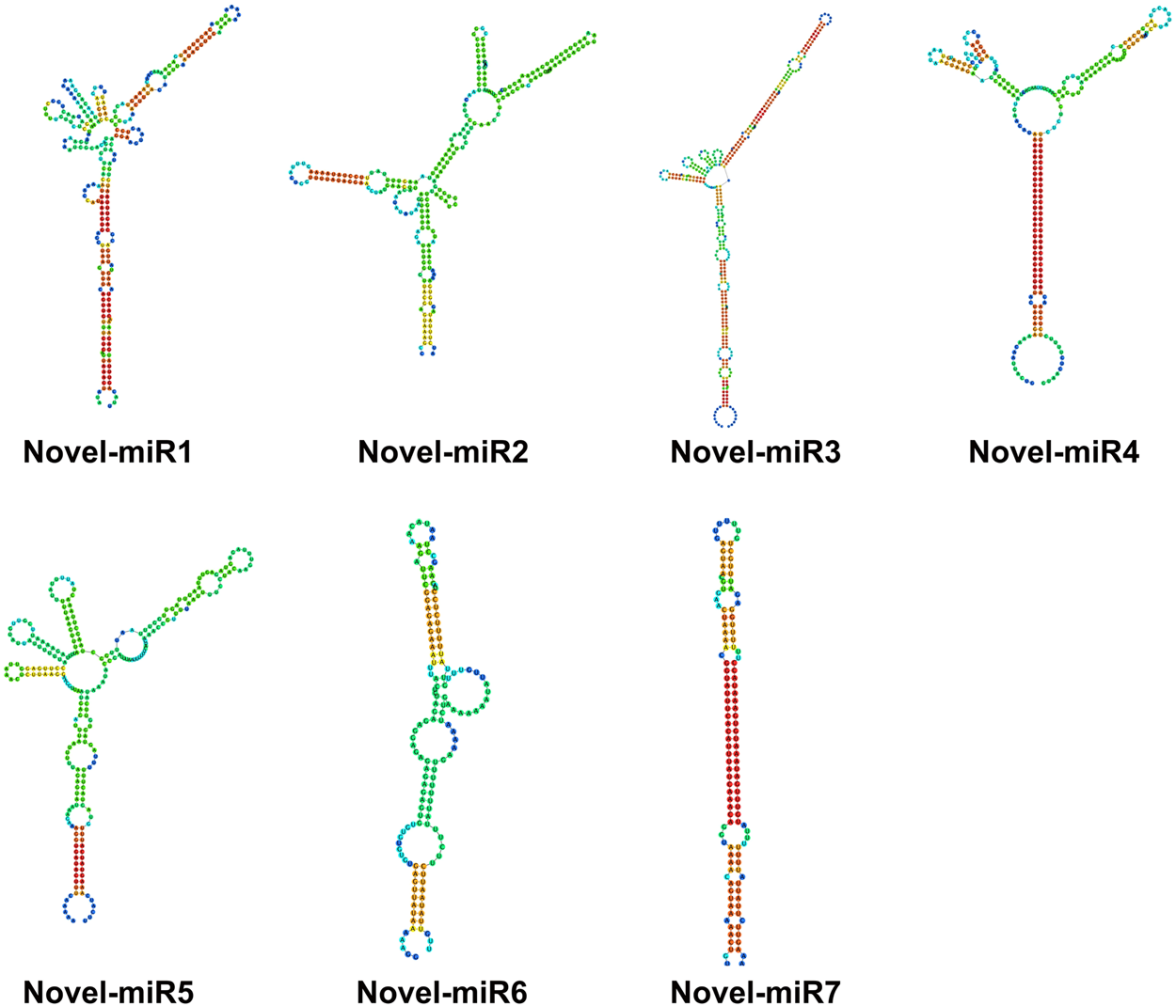
The secondary structures of candidate novel miRNAs.

**Figure 13:**
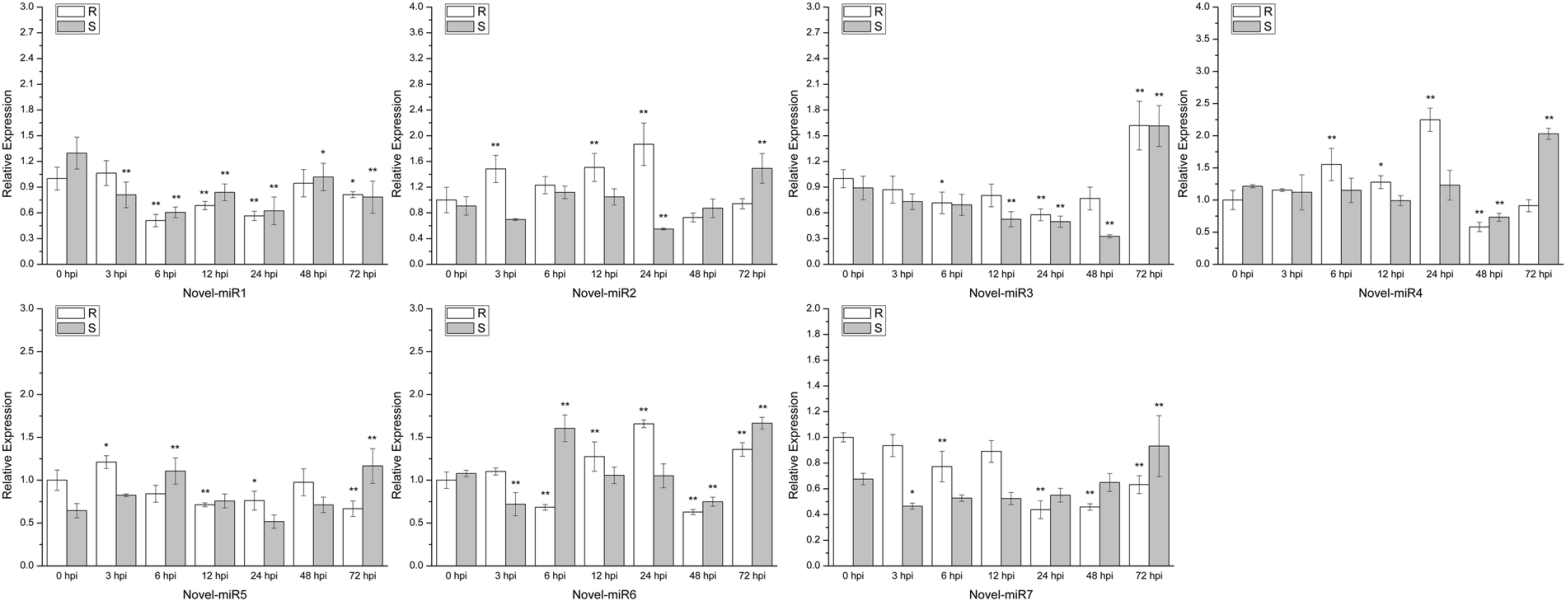
Expression analysis of candidate novel miRNAs and targets at 0, 3, 6, 12, 24, 48 and 72 hpi (hours post-inoculation) using the 2^−ΔΔCt^ method. Data are means (±SD) of three biological replicates per variety. Significance was determined by Duncan’s multiple range test, and is represented by *(P ≤ 0.05) and **(P ≤ 0.01). R = resistant variety. S = susceptible variety.

We used the MIREAP platform to predict new small miRNAs and the RNAfold web server to describe their secondary structures. The previously validated DEMs were used as the target genes to identify potential novel miRNAs, and we identified 7 novel potential miRNAs (Table 6). The secondary structures predicted for these candidate novel miRNAs are shown in Fig. 12. These novel miRNAs were also tested by qRT-PCR, and their expression patterns were analyzed with their targets (Fig. 13). In the resistant variety, the main reduction in Novel-miR1 occurred at 6–24 hpi, while in the susceptible variety, its expression was noticeably reduced after the pathogen infection. In the resistant variety, Novel-miR2 was significantly increased after the *C. cassiicola* infection and then recovered to the normal level at 48 hpi; however, in the susceptible variety, Novel-miR2 was significantly decreased at 24 hpi and increased at 72 hpi. The expression of Novel-miR3 was significantly decreased at 24 hpi but markedly increased at 72 hpi in Jinyou 38, while in the susceptible variety, its expression was obviously decreased after 24 hpi and then significantly increased at 72 hpi. The expression of Novel-miR4 was significantly up-regulated at 6 and 24 hpi and obviously down-regulated at 48 hpi in the resistant variety, but in the susceptible variety, its expression was markedly decreased at 48 hpi and significantly increased at 72 hpi. The expression of Novel-miR5 fluctuated gently after the pathogen infection in both lines. The expression of Novel-miR6 significantly decreased at 6 and 48 hpi and increased at 12, 24 and 72 hpi in Jinyou 38, while in Ludixianfeng, its expression was significantly decreased at 3 and 48 hpi and obviously increased at 6 and 72 hpi. The expression of Novel-miR7 was significantly decreased at 6, 24, 48 and 72 hpi in the resistant variety; however, in the susceptible variety, its expression fluctuated greatly after the pathogen infection and significantly increased at 72 hpi.

**Table 6:** Novel miRNAs and their targets.

MicroRNA	Mature sequence	Targets ID	Targets description
Novel-miR1	CUCUUUGUUGACUUUGAAUUCGAG	Cucsa.261210	4-coumarate: CoA ligase
Novel-miR2	UUCGAAAUGUAAAACUAAAAGTGU	Cucsa.089960	S-adenosylmethionine decarboxylase
Novel-miR3	UUAAUAUAUUGUAAUAUGACCGUU	Cucsa.153390	Peroxidase
Novel-miR4	AUACUCUAGAACAAUCUCUCU	Cucsa.147540	Phosphoenolpyruvate carboxylase
Novel-miR5	AUAGUGGAAAGAAAUAUGAGAUU	Cucsa.017550	Pleiotropic drug resistance protein
Novel-miR6	UGAGUGUGUGUGUGUGAGAG	Cucsa.092350	Xyloglucanendotransglucosylase/hydrolase family protein
Novel-miR7	GACAAAAUGGACAAACUAUUUAC	Cucsa.124480	Phenylalanine ammonia-lyase

## Discussion

In plants, reactive oxygen species (ROS) accumulation and lignin deposition are two early defense responses to pathogen infection^29^. In a previous study, ROS began to increase in the leaves of both cucumber varieties at 6 hpi, and a significant increase was observed at 24 hpi. During the early stage of pathogen infection (6–12 hpi), the ROS content in the resistant variety was relatively higher, indicating that the resistant variety could have a significant hypersensitive response to resist the invasion of pathogens more effectively. At 12 hpi, both cucumber varieties showed lignin deposition, which gradually increased over time. Similarly, the lignin deposition was more obvious in the resistant variety. These results suggested that the resistant variety responds earlier to *C. cassiicola* than the susceptible variety. According to the ITS sequence PCR results, the target bands were detected in both varieties at 6 hpi. In summary, we conclude that the response of cucumber to *C. cassiicola* occurs during the early stage of infection. Therefore, we used the resistant variety for RNA-seq to search for genetic resources related to *C. cassiicola* resistance, and samples at 0, 6 and 24 hpi were used for sequencing.

We analyzed the transcriptomes and miRNAs in the cucumber cultivar Jinyou 38 after inoculation with *C. cassiicola*. In this study, we identified many DEGs in the two groups (0 hpi vs 6 hpi and 6 hpi vs 24 hpi). According to the results of GO term and KEGG pathways enrichment analysis, the secondary metabolism-related genes were strongly induced in Jinyou 38 after *C. cassiicola* infection (Figs 7 and 8). There were 146 DEGs in the intersection of the two groups (0 hpi vs 6 hpi and 6 hpi vs 24 hpi). To further explore the gene expression pattern differences between the resistant and susceptible cucumber cultivars challenged with *C. cassiicola*, we selected 17 candidate genes for qRT-PCR assays (Fig. 10).

Plant hormones are active substances that can regulate the physiological response to environmental factors^30^. In plants, polyamine plays a role in defense signal transduction after pathogen infection^31^. *S-adenosylmethionine decarboxylase* (Cucsa.089960) is a key regulatory gene in the polyamine biosynthesis pathway^32,33^. The expression of *S-adenosylmethionine decarboxylase* was significantly up-regulated at 3 hpi, but no significant difference in expression was observed between the varieties. *Gibberellin 3-oxidase* (Cucsa.004570) can regulate the bioactivity of gibberellin, which plays an important role in plant responses to biotic and abiotic stress^34^. In the resistant variety, the expression of *gibberellin 3-oxidase* (Cucsa.004570) and *gibberellin regulated protein* (Cucsa.343030) was up-regulated earlier and to a higher degree. Thus, the gibberellin signaling pathway may be related to the response to *C. cassiicola* in cucumber.

MYB transcription factors play a key role in the plant defense response and can activate certain plant *PR* genes, leading to an increase in resistance to stress^35^. The expression of *MYB transcription factor* (Cucsa.121500) significantly increased after 24 hpi in both varieties, but during the early infection stage, its expression was higher in the resistant variety.

**Figure 14:**
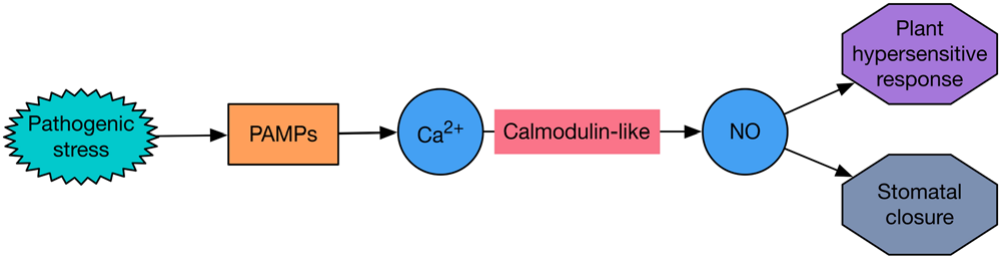
Ca^2+^ signals pathway of cucumber in response to *C. cassiicola*.

*C. cassiicola* is an obligate fungus that requires host energy for its nutrition; therefore, plants may inhibit the spread of pathogens by reducing energy metabolism. During the early stage of infection, *phosphoenolpyruvate carboxylase* (Cucsa.147540) and *carbonic anhydrase* (Cucsa.339710) were significantly decreased. Both genes are associated with photosynthesis^36,37^, supporting the hypothesis that hosts may limit energy metabolism to resist the pathogen. The calcium signaling pathway plays an important role in plant stress resistance because Ca^2+^ can activate and regulate the expression of downstream genes as a secondary messenger^38^. Calmodulin-like is a key factor in plant-pathogen interactions. When a plant is subjected to pathogenic stress, pathogen-associated molecular patterns (PAMPs) produce specific Ca^2+^ signals, and calmodulin-like mediates NO signaling to induce the plant hypersensitive response (Fig. 14). The significant increase in *calmodulin-like* (Cucsa.254730) expression first occurred at 6 hpi in the resistant variety, while in the susceptible variety, the increase occurred at 24 hpi. Therefore, calmodulin-like in the resistant cultivar could respond to stress and regulate the downstream pathway earlier.

We also identified several defense-related genes, most of which were associated with the cell wall and reactive oxygen scavenging. *Xyloglucan endotransglucosylase/hydrolase family protein* (Cucsa.092350) is a cell wall-modified protein with the ability to loosen cell walls^39^. These genes have been recently shown to be involved in multiple growth regulation processes, including cell extension and the stress response^40,41^. In this study, these genes responded earlier to stress in the resistant varieties. We speculate that these genes may provide resistance to pathogen stress by altering the toughness of the cell wall. Chitinase can hydrolyze chitin in the cell wall of the pathogen, and the digested oligomeric product can also induce further defense as a signal molecule^42^. Unfortunately, the *chitinase* (Cucsa.152090) expression patterns were similar in both varieties. The *PR protein* is the terminal gene of the plant defense pathway and can induce resistance in the response to pathogens^43^. Thaumatin is a protein with multi-biological activity and important function in the plant defense class of sweet protein, belonging to the PR proteins^44^. The eukaryotic aspartyl protease family protein is an important hydrolase that is used to synthesize specific disease resistance-related proteins and hydrolyze the proteins secreted by invading pathogens^45^. Under pathogen stress, many ROS in plant outbreaks can improve plant resistance but can also destroy the organizational structure of the plant. Plants often produce peroxidase to remove excess ROS, thereby relieving the damage caused by oxidative stress to the plant^46^. After 12–24 hpi, the expression of *PR protein* (Cucsa.133220), *thaumatin* (Cucsa.302870), *aspartyl protease family protein* (Cucsa.043900) and *peroxidase* (Cucsa.153390) in both varieties was nearly identical, and after 12–24 hpi, their abundances were at a very high level. We also speculate that these genes play an important role in resistance to *C. cassiicola*, although no difference was observed between the varieties.

**Figure 15:**
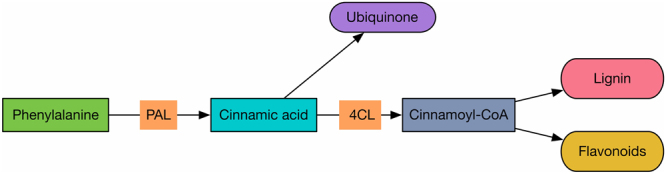
Phenylpropanoid synthesis pathway of cucumber in response to *C. cassiicola*.

Secondary metabolites are key factors inducing systemic resistance^47^. The pleiotropic resistance protein is an ATP-binding cassette (ABC) type transporter that plays an important role in the regulation of secondary metabolism and environmental adaptation^48^. The significant accumulation of *pleiotropic resistance protein* (Cucsa.017550) in the resistant variety appeared at 24 hpi, but in the susceptible variety, this accumulation occurred at 48 hpi. Cytochrome P450 can catalyze certain secondary metabolites, such as indole, phenylpropane, phytohormones and alkaloids, to improve plant immunity^49,50^. In our study, the expression of *cytochrome P450 CYP2* (Cucsa.342000) was higher in the resistant variety, particularly at 24 hpi. Phenylalanine ammonia-lyase is closely related to plant stress resistance as follows: phenylalanine ammonia-lyase acts as the initial enzyme in flavonoid biosynthesis and promotes the synthesis of flavonoids, thereby allowing plants to resist external stress^51^. In addition, 4-coumarate: CoA ligase is an important enzyme in the phenylpropanoid pathway in plants that affects the synthesis of lignin^52^. These two genes belong to the pathway of phenylpropanoid synthesis. As shown in Fig. 15, phenylalanine ammonia-lyase (PAL) first catalyzes phenylalanine to cinnamic acid. Cinnamic acid is involved in the synthesis of ubiquinone and can be catalyzed by 4-coumarate: CoA (4CL) ligase to form cinnamoyl-CoA. Cinnamoyl-CoA is then involved in the synthesis of lignin and flavonoids. The expression patterns of *phenylalanine ammonia-lyase* (Cucsa.124480) and *4-coumarate: CoA ligase* (Cucsa.261210), which are the two key genes in this pathway, were very similar. After 12 hpi, the expression levels of these genes were significantly higher in the resistant variety. Therefore, under the stress of *C. cassiicola* infection, secondary metabolism in cucumber plays an important role in disease resistance.

The interactions among the miRNAs and their targets can offer a complex mechanism for the response to stress^23,53^. In this study, miRNAs were also sequenced to explore the role of the miRNAs in the regulation of cucumber mRNAs under *C. cassiicola* infection stress. In this study, we identified 34 DEMs belonging to 17 families and 7 novel miRNAs with precursors using high-throughput sequencing techniques and bioinformatics analysis. We also predicted many targets for the miRNAs in combination with the transcriptome data. The qRT-PCR results of the candidate miRNAs and targets showed that their expression patterns differed between the cucumber varieties (Fig. 11). In our study, most candidate miRNAs, including miR164d, miR167e, miR171f and miR172c, target various transcription factors, such as NAC, ARF, SCL and AP. In this assay, miR164d targets *NAC domain containing protein* (Cucsa.040380). NAC is a plant-specific transcription factor family that is not only involved in the process of growth and development but also participates in stress responses^54^. In *Jatropha curcas*, NAC can improve plant resistance by regulating hormone signaling. The overexpression of the *NAC* gene can significantly improve the tolerance of *Jatropha curcas* to *Pseudomonas syringae*^55^. Several studies have shown that NAC transcription factors are related to lignin synthesis^56,57^. The target gene for miR167e was *ARF*, which can bind to auxin response elements. ARF can activate the downstream hormone signaling pathway to promote the expression of defense genes in plants. These expression products can be transmitted as signal molecules to allow plants to perceive signals and respond^58^. We predicted that the *GRAS family transcription factor* (Cucsa.320850) is cleaved by miR171f. GRAS is a plant-specific transcription factor that plays an important role in signal transduction and hormone regulation^59^. Our results show that the target of miR172c is APETALA, which is an ethylene-responsive transcription factor. APETALA plays a very important role in plant growth and development, including the development of flower organs, fruit development, seed formation and stress response^60^. Due to its importance in plants, the regulation of miR172c to the target gene *APETALA* is essential for plants to resist pathogens and improve their resistance. The target gene for miR390a is *LRR-RLK* (Cucsa.164200). LRR-RLK proteins constitute a variety of transmembrane receptors involved in biological functions in plants, such as growth and development and response to biological and abiotic stress^61,62^. Unfortunately, in our assays, the LRR-RLK proteins may be inhibited by miR390a; thus, their expression declined in both varieties. We hypothesize that the expression of miR390a can be reduced to increase the expression of LRR-RLK, which may help improve the resistance of the host to *C. cassiicola*. The target gene of miR395c is *ATP sulfurylase* (Cucsa.254710), which is the first enzyme in the process of sulfate assimilation. Sulfur is not only involved in the synthesis of sulfur-containing amino acids and proteins but is also an important component of many enzymes and cofactors. Sulfur is also related to metabolic substances produced by plants under stress^63,64^. miR396b is a conserved class of miRNAs in plants. In this assay, the target of miR396b is *anthranilate phosphoribosyltransferase* (*APE*), which is related to tryptophan synthesis^65^. In plants, the endogenous jasmonic acid (JA) biosynthesis, plant signal transduction pathway and terpenoid indole alkaloid (TIA) pathway were triggered by tryptophan, which can regulate plant responses to stress^66^. *Plantacyanin* (Cucsa.077170) is the target gene of miR408. In chloroplast photosynthesis, plantacyanin is a copper-containing protein that serves as an electron transporter. Plantacyanin is indirectly involved in the removal of ROS in plants; therefore, plantacyanin is closely related to plant resistance^67^.

We identified seven novel miRNAs that targeted several previously validated DEMs (Table 6). These targets, such as 4-coumarate: CoA ligase, pleiotropic drug resistance protein, and phenylalanine ammonia-lyase, are mainly associated with secondary metabolism. Since Novel-miR1 and Novel-miR7 can cleave *4-coumarate: CoA ligase* and *phenylalanine ammonia-lyase* separately, we hypothesized that if Novel-miR1 and Novel-miR7 were decreased, the expression of genes in the phenylpropanoid and lignin biosynthesis pathway would be promoted, which can greatly improve the resistance of cucumber to *C. cassiicola*.

**Figure 16:**
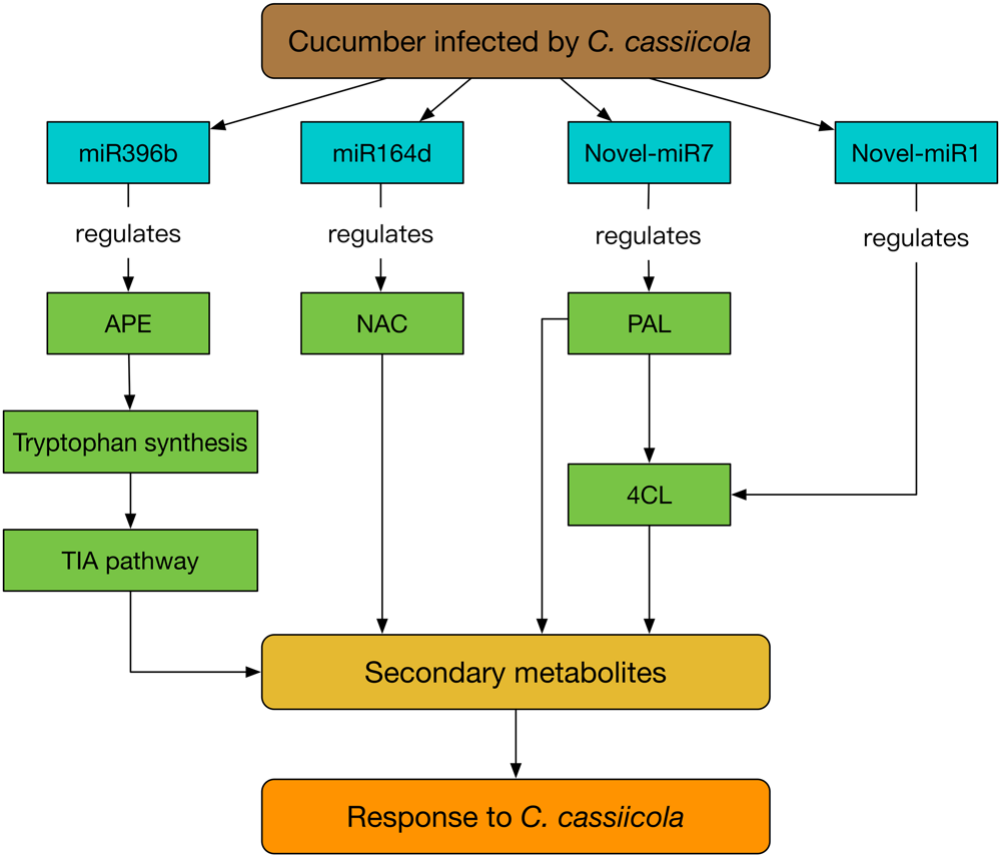
Possible mechanism of secondary metabolism affecting the response to *C. cassiicola*.

The experimental results showed that the candidate miRNAs are mainly involved in the regulation of secondary metabolism-related genes, so the secondary metabolism-related defense responses in resistant variety occured earlier and faster. These results combined with our previous data indicated that secondary metabolism plays an important role in cucumber resistance to *C. cassiicola* infection. Based on the above analysis, we summarized these regulatory pathways related to secondary metabolism (Fig. 16). Secondary metabolism was regulated by the interaction of these miRNAs and targets, which could affect the resistance of cucumber to TLS.

## Conclusions

The response time is critical for cucumber resistance to *C. cassiicola* infection, as the results of ROS and lignin staining suggested that the resistant variety Jinyou 38 showed a faster response to pathogen stress. At the early stages of infection, we obtained many DEGs from the resistant variety that were involved in the response to *C. cassiicola*. The functional analyses showed that these DEGs were mainly concentrated in secondary metabolism. We performed qRT-PCR assays on DEGs related to plant hormone, transcription factor, metabolic correlation, Ca^2+^ signaling pathway, secondary metabolism and defense genes. The results showed that the resistance of Jinyou 38 may be mainly attributed to secondary metabolism. In addition to transcriptome sequencing, this study also sequenced known miRNAs and predicted novel miRNAs in cucumber that contribute to the interaction between the host and *C. cassiicola*. By analyzing the functions of these targets, we found that several known and novel miRNAs may mediate secondary metabolism. Combined with the transcriptome and miRNA data, the results of this study were used to summarize the mechanism of secondary metabolism in the resistance of cucumber to *C. cassiicola*. Overall, these results provide a new theoretical basis for studies investigating resistance mechanisms and key resistance genes against cucumber TLS at the molecular level.

## Methods

### Plant growth and *C*. *cassiicola* inoculation

The cucumber varieties used in the experiments were Jinyou 38 (preliminary experiments showed that this variety was resistant to TLS) and Ludixianfeng (TLS susceptible), which were planted in a greenhouse at 28 °C under 16:8 light/dark cycles. *C. cassiicola* was obtained from the Agricultural Culture Collection of China. The true leaves of 6-week-old plants were sprayed with spore suspensions (2 × 10^5^ sporangia/ml). Then, the inoculated materials were kept at 100% relative humidity to ensure spore germination. The inoculated leaves were harvested at seven time points (i.e., 0, 3, 6, 12, 24, 48 and 72 h post-inoculation), frozen in liquid nitrogen and stored at −80 °C. All experiments were performed in three independent replicates for each time point.

### PCR assay for the detection of *C*. *cassiicol*

The DNA of the inoculated leaves was extracted using the Plant Genomic DNA Kit (Tiangen, Beijing, China). *C. cassiicola* internal transcribed space (ITS) primers were used to detect whether the pathogen entered the host. For the qPCR analysis, the following cycling parameters were used: 1 hold at 94 °C for 5 min, 35 cycles at 94 °C (30 s), 60 °C (15 s), 72 °C (1 min), and 72 °C (10 min). The PCR products were detected by agarose gel electrophoresis.

### Histochemical staining

Lignified tissues were stained with 1% phloroglucinol solution (Maclin, Shanghai, China). First, the plant leaves were soaked in stationary liquid containing 95% ethyl alcohol and glacial acetic acid (1:1, v/v) for 24 h, rinsed with distilled water, vacuum treated in saturated aqueous solution of chloral hydrate for 10 min, and stored at room temperature for two or three days until they became transparent.

DAB staining was performed to detect H_2_O_2_ in the inoculated plants. The cucumber leaves were soaked in a DAB (Sigma, MO, USA) solution (1 mg/ml) for 8 h and placed on the shaker (80~100 rpm) for 4–5 h. The leaves were added to the decoloring solution containing absolute ethyl alcohol, glacial acetic acid and glycerol (3:1:1, v/v/v) and heated until the tissues were transparent for observation.

The O_2_^−^ of the infected leaves was observed by performing NBT staining. The samples were infiltrated with a 0.1% NBT (Ameresco, OH, USA) solution for 4 h and subjected to a vacuum treatment for 15 min. Decolorization is essentially the same as that described above.

### mRNA-seq and analysis

The three time points of the inoculated leaves were defined as follows: 0, 6, and 24 hpi. Leaves at the three time points were selected for transcriptome sequencing using an Illumina NextSeq 500 platform (Personal Biotechnology, Shanghai, China). The RNA of the three samples at each time point were pooled in equal volumes (the concentrations were adjusted to 10 nM).

The transcripts were annotated via BLAST against the cucumber genome database (https://phytozome.jgi.doe.gov/pz/portal.html). The expression of each gene was calculated according to the reads per kilo bases per million reads (RPKM). The expression level was calculated according to the baseMean value, which is the sequencing depth of each transcript normalized to the library size. The baseMean value and *P*-value of the genes were calculated by the DESeq platform. Genes with expression changes greater than twofold between two points were considered DEGs (*P*-value < 0.05). All DEGs were analyzed with eggNOG functional categories (http://eggnog.embl.de/), GO enrichment (http://www.geneontology.org/) and KEGG enrichment (http://www.genome.jp/kegg/)^68–71^.

### MicroRNA-seq and analysis

The RNA samples used for the miRNA-seq were the same as those used for the mRNA-seq. The sequencing of the miRNAs was performed by Personalbio using the Illumina NextSeq500 system. The expression quantity was calculated according to the transcripts per million (TPM). To identify the known miRNAs, the clean reads were BLASTed against the miRNA database miRbase 21.0 (http://www.mirbase.org/). Because miRBase contains few cucumber miRNA sequences, we used melon data as a reference. We also analyzed the miRNA families and conservation in miRbase. The DEMs were screened according to the difference in expression |fold change| >2). The RNAfold web server (http://nhjy.hzau.edu.cn/kech/swxxx/jakj/dianzi/Bioinf4/miRNA/miRNA1.htm) was used to map the pre-miRNAs. For sequences that were not annotated to any information, we predicted new miRNAs using mireap (http://sourceforge.net/projects/mireap/). Since the miRNAs are primarily bound to the target site by complementary pairing, based on the transcriptome data, the targets of the mature miRNA sequences were identified using miranda^72^ (http://www.microrna.org/microrna/home.do).

### Quantitative real-time RT-PCR assay

RNA was extracted using the RNAprep Pure Plant Kit (Tiangen, Beijing, China) and synthesized into cDNA using the FastQuant RT Kit (with gDNase) (Tiangen, Beijing, China). Quantitative real-time RT-PCR (qRT-PCR) was conducted using a SuperReal PreMix Plus Kit (SYBR Green) (Tiangen, Beijing, China). The qRT-PCR was performed on a LightCycler 480 (Roche Molecular Systems, CA, USA). Cucumber *Actin* was used as the reference gene to normalize the data. To validate the presence and relative expression of the miRNAs obtained from the sequencing, the known and novel miRNAs were assayed by qRT-PCR. The reverse transcription reaction was performed using a miRcute miRNA First-Strand cDNA Synthesis Kit (Tiangen, Beijing, China). The qRT-PCR was performed using the miRcute miRNA qRT-PCR Detection Kit (SYBR Green) (Tiangen, Beijing, China), and U6 snRNA was used as the internal control. The relative expression was calculated according to the 2^−ΔΔCt^ method^73^, and the standard deviation was calculated using three biological replicates. The primers used in the experiment were synthesized by GENEWIZ (Suzhou, China) and are listed in Supplementary Table S5.

### Data availability

The mRNA raw data were deposited in the NCBI Sequence Read Archive (SRA) with the accession number SRP117262; the small RNA raw data were deposited in the NCBI Sequence Read Archive (SRA) with the accession number SRP117230.

## Electronic supplementary material

Dataset 1

Dataset 2

Dataset 3

Dataset 4

Dataset 5

Dataset 6
